# The incidence of early onset colorectal cancer in Aotearoa New Zealand: 2000–2020

**DOI:** 10.1186/s12885-024-12122-y

**Published:** 2024-04-12

**Authors:** Oliver Waddell, John Pearson, Andrew McCombie, Harriet Marshall, Rachel Purcell, Jacqueline Keenan, Tamara Glyn, Frank Frizelle

**Affiliations:** 1 Department of Surgery and Critical Care, University of Otago Christchurch, 36 Cashel St, Christchurch central, Christchurch, New Zealand; 2 Biostatistics and Computational Biology Unit, University of Otago Christchurch, Christchurch, New Zealand; 3 Department of General Surgery, Te Whatu Ora Health New Zealand, Christchurch, New Zealand

## Abstract

**Background:**

The incidence of early-onset colorectal cancer (EOCRC), diagnosed before age 50, has been rising in many countries in the past few decades. This study aims to evaluate this trend in Aotearoa New Zealand and assess its impact on Māori.

**Methods:**

Crude incidence and age-standardized incidence of colorectal cancer (CRC) was analyzed from all new cases from the Aotearoa New Zealand national cancer registry for the period 2000–2020. Trends were estimated by sex, ethnicity, age group and location of cancer and projections made to 2040.

**Results:**

Between 2000 and 2020, there were a total of 56,761 cases of CRC diagnosed in Aotearoa New Zealand, 3,702 of these being EOCRC, with age-standardized incidence decreasing significantly (*P* = 8.2 × 10^− 80^) from 61.0 to 47.3 cases per 100,000. EOCRC incidence increased on average by 26% per decade (incidence rate ratio (IRR) 1.26, *p* = < 0.0001) at all sites (proximal colon, distal colon and rectum), while the incidence in those aged 50–79 years decreased on average by 18% per decade (IRR 0.82, *p* = < 0.0005), again across all sites. There was no significant average change in CRC incidence in those over 80 years. In Māori, there was no significant change in age-standardized incidence. There was however a significant increase in crude incidence rates (IRR 1.28, *p* = < 0.0005) driven by significant increases in EOCRC (IRR1.36, *p* = < 0.0005). By 2040, we predict the incidence of EOCRC will have risen from 8.00 to 14.9 per 100,000 (6.33 to 10.00 per 100,000 in Māori). However, due to the aging population an estimated 43.0% of all CRC cases will be diagnosed in those over 80 years of age (45.9% over 70 years of age in Māori).

**Conclusion:**

The age-standardized incidence of CRC from 2000 to 2020 decreased in Aotearoa New Zealand, but not for Māori. The incidence of EOCRC over the same period continues to rise, and at a faster rate in Māori. However, with the ageing of the population in Aotearoa New Zealand, and for Māori, CRC in the elderly will continue to dominate case numbers.

**Supplementary Information:**

The online version contains supplementary material available at 10.1186/s12885-024-12122-y.

## Background

Colorectal cancer (CRC) is the second most common cancer in Aotearoa New Zealand in both men and women; it is second only to prostate and breast cancers, with 3,515 colorectal cancers diagnosed in Aotearoa New Zealand in 2020. It remains the second highest cause of cancer death with over 1,200 people dying each year as a result [1]. While the age-standardized rate of CRC in Aotearoa New Zealand is slowly declining, the rate of early-onset colorectal cancer (EOCRC), defined as CRC diagnosed in adults under the age of 50 years, is rising [2]. This pattern is not confined to Aotearoa New Zealand, with increases in rates of EOCRC being observed in at least 18 other countries around the world [3]. This trend is occurring independently of trends in older patients and has been reported in countries where the rates of CRC in older adults are rising, stable or falling [3, 4]. Bowel cancer screening was initially trialled in parts of Aotearoa New Zealand starting from 2012, with national bowel cancer screening being widely available from 2021 for those aged 60–74 years. This will be having an impact on CRC incidence and survival in these age groups, however, will not be impacting incidence in the younger unscreened population.

The clinical characteristics of EOCRC can differ from late-onset colorectal cancer (LOCRC, diagnosed over 50 years of age), with EOCRC more likely to be left sided (sigmoid and rectum), have higher rates of mucinous or signet ring histology, be poorly differentiated, and present with advanced (stage 3 or 4) disease [5, 6]. Despite these differences, a recent meta-analysis found no overall difference in cancer specific survival between early-onset and late-onset disease [7]. 

This study describes the population trends in CRC incidence in Aotearoa New Zealand from 2000 to 2020, analyzed by sex and age group, and investigates trends in incidence rates in Māori. This study also makes predictions for the near future, should the expected aging of the population continue.

## Methods

All new cancers except basal cell and squamous cell carcinomas of the skin, diagnosed in Aotearoa New Zealand are registered with the New Zealand Cancer Registry (NZCR) [8]. Prior to 1995, this data was incomplete but since 1995, collection of all cancers, except for non-melanotic skin cancers, has been compulsory, giving rise to a high-quality dataset of all cancers diagnosed in Aotearoa New Zealand. Demographic data, date of diagnosis, tumour site, tumour type, morphology, grade of disease and staging are recorded. For CRC, tumour site was divided into proximal (caecum, ascending colon, hepatic flexure, transverse colon, splenic flexure), distal (descending colon, sigmoid colon) and rectal (rectosigmoid, rectal). Other sites (appendix, unspecified sites, and anus) were excluded. Histological type was limited to adenocarcinoma only.

We have made projections, see statistical methods, to 2040 based on the 2000–2020 data, using Statistics New Zealand’s most recent median growth and immigration population projections, which have a 2022 base year (hence there is no 2021 projection) [8]. Ethnicity information was taken from the NZCR, and is determined based on the Ministry of Health ethnicity data protocols, and reports on prioritised Māori ethnicity. These use self-reported ethnicity data from the census, as well as from National health index (NHI), the mortality collection and hospital discharge data in the national minimum data set [9]. Māori population figures and projected populations were provided by Statistics New Zealand. These figures are determined using information from the census and incorporating a components-based method to estimate the changes in Māori population numbers [10–12]. 

### Ethics approval

#### Ethical approval

was obtained from the University of Otago Health Ethics Committee (UOHEC), (HD23/044), and University of Otago, Māori health advancement Komiti. The need for informed consent was waived by the institutional review board, the University of Otago Health Ethics Committee (UOHEC). Furthermore, data from cancer registry is allowed to be used for the purpose of cancer incidence studies as stated in the New Zealand Cancer Registry Act 1993 [13]. Our research processes and reporting are aligned with and have been guided by the CONSIDER statement [14] and we have completed the CONSIDER checklist (Supplementary Table 1).

### Statistics

To calculate ASIR, crude incidence rates were age-standardized to the European Standard population (ESP) 1976 [15], (or the Māori population in 2000). ESP was used as it is the international standard closest to the Aotearoa New Zealand age structure, and it also allows for international comparison. Incidence rates for total ethnicity and Māori ethnicity were modelled separately using Poisson regression, by offsetting cases by the log of the population, with covariates for age group (three groups), sex (dichotomous) and location (proximal, distal, rectal). Age groups for total ethnicity were split at 50 and 80 years of age, and for Māori ethnicity were split at 50 and 70 years of age, with the oldest age group containing approximately 3% of the population for both total and Māori ethnicity. This was done in an attempt to account for the different age distribution of the Māori population. Time-dependent co-variables were time (decades since 2000), time by sex, and all interactions of time, age group and location for total ethnicity, but only time and time by age group for Māori ethnicity as there was no statistical support for further terms. All model terms were tested for significance with likelihood ratio tests; no other interactions significantly improved fit using the likelihood ratio test (*P* > 0.05). The models showed no evidence of lack of fit, *P* > 0.408, (0.269 Māori), over dispersion or inferior fit to a Negative Binomial model, Log Likelihood test df = 29, *P* = 0.795.(df = 18 *P* > 0.99). Autocorrelation plots revealed no evidence of correlation with lagged variables (all *r* < 0.5.) Confidence intervals for model parameters were calculated using robust errors and those for incidence rate ratios (IRRs) were calculated using the delta method. Projections for age-standardized incidence were performed by refitting models with age grouped by decade, with the first two decades collapsed to avoid small cell counts, with populations given by Statistics New Zealand’s median population growth projections for the years 2022 to 2040. We assumed ongoing linear changes in incidence for each age group. Indirect age standardisation was performed post hoc to the European standard population for the total population and the Māori population for Māori ethnicity, with age groups collapsed as in the model. Projections to 2040 were made by using the same models to estimate crude incidence at later populations.

## Results

The average population in Aotearoa New Zealand over the period of the study increased from 3.83 million to 5.09 million. Between 2000 and 2020, there were a total of 56,761 cases of CRC diagnosed in Aotearoa New Zealand; 3,702 (6.5%) of these were EOCRC.

There was no significant change in crude incidence of CRC from 2000 to 2020, (IRR = 1.00, 95% CI (0.98,1.01), *P* = 0.54). However, there was a significant reduction in the age-standardised incidence, from 61.0 per 100,000 to 47.3 per 100,000, over the same period at an average rate of 87.7% per decade (*P* = 8.2 × 10^− 80^), reflecting our aging population (Table 1). Moreover, these changes were unevenly distributed across age groups, with EOCRC incidence increasing from 4.41 per 100,000 in 2000 to 8.03 per 100,000 in 2020, an average increase of 26% per decade (IRR 1.26, *p* = < 0.0005). The proportion of total CRC that were diagnosed in those under the age of 50 increased from 5.3% in 2000 to 8.6% in 2020. The incidence of CRC in patients aged 50–79 years reduced from 193.4 per 100,000 to 133.8 per 100,000 (IRR 0.82, *p* = < 0.0005) whereas in those aged 80 years and over there was no significant overall change in incidence (IRR 0.99 (95% CI 0.96, 1.02) *p* = 0.632) (Tables 1 and 2). We have also included a more detailed breakdown of proportions of EOCRC cases occurring in narrower age brackets in Supplementary Table 4.

**Table 1:** Colorectal cancer incidence in Aotearoa New Zealand

		2000		2010		2020	
		*Total*	*Māori*	*Total*	*Māori*	*Total*	*Māori*
**Population**		3.83 M	0.60 M	4.37 M	0.66 M	5.09 M	0.85 M
**New registrations**		2,344	84	2,727	146	3,142	244
**Incidence**		61.15	14.03	62.40	21.99	61.78	28.54
**EOCRC**							
Population		2.81 M	530 K	3.03 M	559 K	3.03 M	679 K
cases		124	9	179	25	270	43
Incidence		4.41	1.70	5.75	4.48	8.03	6.33
**Midlife**							
Population		0.92 M	58 K	1.19 M	87 K	1.19 M	143 K
cases		1765	46	1882	81	2075	132
Incidence		193.41	79.35	156.46	93.56	133.78	92.24
**Older**							
Population		107 K	11 K	151 K	19 K	186 K	33 K
cases		445	29	700	40	805	69
Incidence		419.58	255.28	456.51	214.59	431.67	208.90
**Age Standardised Incidence - Total Ethnicity standardized to European Standard population**							
		*ASI*	*(95% CI)*	*ASI*	*(95% CI)*	*ASI*	*(95% CI)*
Total		61.0	(58.5,63.6)	53.6	(51.5,55.6)	47.3	(45.6,49.0)
Sex	Female	56.0	(52.6,59.3)	47.6	(45.0,50.3)	39.5	(37.3,41.6)
	Male	67.3	(63.4,71.2)	60.2	(57.0,63.4)	55.9	(53.2,58.5)
Location	Proximal	22.4	(20.9,24.0)	21.8	(20.5,23.1)	17.0	(16.1,18.0)
	Distal	16.3	(20.9,17.6)	14.1	(20.5,15.1)	13.3	(16.1,14.2)
	Rectal	22.3	(15.0,23.9)	17.7	(13.0,18.9)	16.9	(12.4,17.9)
**Age Standardised Incidence - Māori Ethnicity standardized to Māori population 2000**							
Total		16.6	(13.2,19.9)	19.8	(16.8,22.9)	15.2	(13.0,17.3)
Sex	Female	14.4	(10.1,18.8)	15.7	(11.9,19.5)	12.9	(10.1,15.7)
	Male	19.0	(13.7,24.4)	24.6	(19.6,29.5)	17.7	(14.3,21.1)
Location	Proximal	4.7	(0.0,6.5)	6.9	(0.0,8.7)	4.1	(0.0,5.2)
	Distal	4.8	(3.0,6.6)	4.3	(5.1,5.8)	4.3	(3.0,5.5)
	Rectal	7.0	(3.0,9.2)	8.6	(2.9,10.6)	6.7	(3.2,8.2)

Incidence is per 100,000 population. Age Standardized Incidence ASI, and 95% Confidence intervals CI Early onset CRC < 50 years, older age begins at 80 years for total ethnicity and 70 years for Māori ethnicity, both approximately 3% of their populations

**Table 2:** Incidence rate ratios

	Total	Māori
	*IRR (95% CI)*	*IRR (95% CI)*
All ages	1.00 (0.98, 1.01)	1.28 (1.21,1.36)***
EOCRC	1.26 (1.20, 1.33)***	1.36 (1.21,1.54)***
Midlife	0.82 (0.81, 0.84)***	0.97 (0.92,1.03)
Older	0.99 (0.96, 1.02)	0.96 (0.89,1.04)

Incidence Rate Ratio over 10 years with 95% confidence intervals. Early onset CRC < 50 years, older age begins at 80 years for total ethnicity and 70 years for Māori ethnicity, both approximately 3% of their populations. *** *P* < 0.0005. Models to calculate IRR include Age, Gender and location of cancer

### Trends in CRC in Māori patients

In 2020, 0.85 M out of 5.09 M (16.7%) New Zealanders were of Māori ethnicity. CRC incidence in Māori increased by 28% per decade (IRR 1.28, *p* = < 0.0005). Contrasting with the total population, there was no material reduction in ASIR in Māori, with ASIR decreasing at an average rate of 0.16 per decade (*p* = 0.754), however this result was somewhat impacted by higher variation in data for Māori. There were significant differences in rates of change by age group, with incidence in Māori aged under 50 years increasing by 36% per decade (IRR 1.36, *p* = < 0.0005), and no significant difference seen in older age groups (Table 2). There was no evidence that incidence rates differed by site of disease or sex in Māori (analysis of deviance *P* > 0.57). Sensitivity analysis showed that there was no material change to conclusions by shifting the older age cut-off from 80 to 70 years, to better accommodate Māori demographics. There was insufficient power to include location in the model for the Māori population.

### Trends by sex and site of disease

**Fig. 1:**
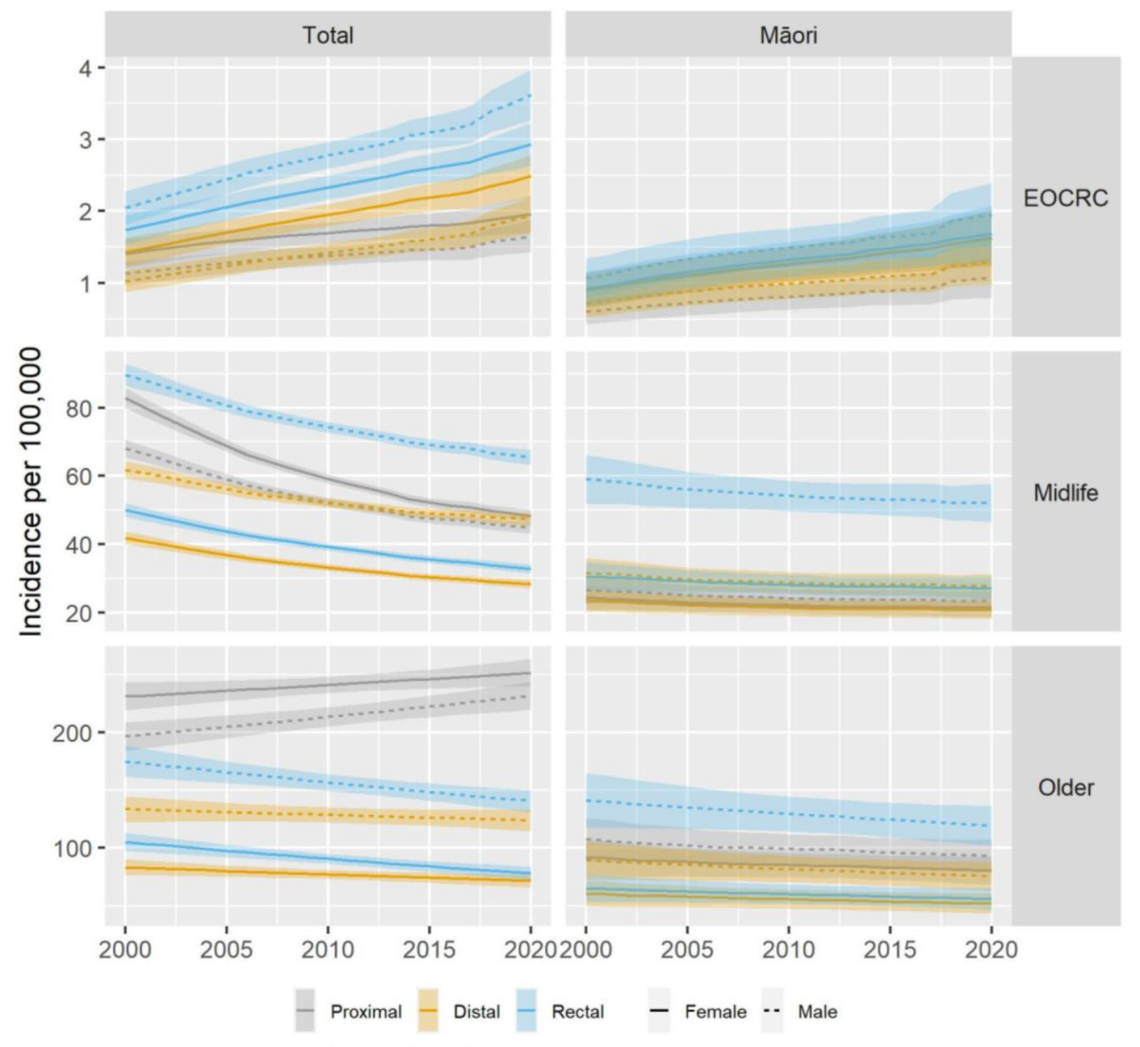
Incidence rate trends for total population and Māori ethnicity. Incidence rates shown by total population and Māori ethnicity by age group, site of tumor and sex. EOCC is less than 50 years old, older age begins at 80 for total ethnicity and 70 for Māori ethnicity. *Note different scale on y-axes for each age group*

In the total population, we observed differences in rates of change when comparing different age groups, sex, and sites of disease. In those aged under 50 years, the largest changes were seen in distal colonic and rectal cancers, with distal colonic cancers rising 29% per decade in women (IRR 1.29, *p* = < 0.0005) and 35% in men (IRR 1.35, *p* = < 0.0005), while rectal cancers in this age group rose by 25% per decade in women (IRR 1.25, *p* = < 0.0005) and 32% per decade in men (IRR 1.32, *p* = < 0.0005), with smaller increases in proximal cancers (IRR in women 1.14, *p* = < 0.05, IRR in men 1.20, *p* = < 0.005). In those aged 50–79 years, there was a significant decrease in incidence across both sexes and each site of disease, with the largest decrease seen in proximal cancers in women (IRR 0.77, *p* = < 0.0005). In those ages 80 + years, there was a more mixed picture; incidence of proximal colonic cancers increased significantly in men by 9% per decade (IRR 1.09, *p* = < 0.0005), while there were significant decreases seen in distal colonic cancers in women (IRR 0.92, *p* = < 0.05), rectal cancers in women (IRR 0.86, *p* = < 0.0005), and rectal cancers in men (IRR 0.90, *p* = < 0.0005) (Table 3; Fig. 1). Full summary of crude incidence rates by year, sex, age, and site of disease for the total population and for Māori can be found in Supplementary Tables 2 and 3.

**Table 3:** Total population Incidence Rate Ratios by sex and site of disease

		Female	Male
*Age (years)*	*Site*	*IRR*	*IRR*
< 50	Proximal	1.14 (1.02,1.28)*	1.20 (1.07,1.35)**
	Distal	1.29 (1.17,1.41)***	1.35 (1.24,1.48)***
	Rectal	1.25 (1.18,1.32)***	1.32 (1.25,1.39)***
50–79	Proximal	0.77 (0.74,0.79)***	0.81 (0.78,0.83)***
	Distal	0.83 (0.79,0.86)***	0.87 (0.84,0.91)***
	Rectal	0.81 (0.79,0.84)***	0.86 (0.84,0.88)***
80+	Proximal	1.04 (1.00,1.08)	1.09 (1.05,1.14)***
	Distal	0.92 (0.87,0.97)*	0.97 (0.92,1.02)
	Rectal	0.86 (0.81,0.90)***	0.90 (0.85,0.95)***

Incidence Rate Ratio over 10 years with 95% confidence intervals.* *P* < 0.05, ** *P* < 0.005, *** *P* < 0.0005

### Projections

Table 4 shows the predicted trends in population, CRC cases, and CRC incidence in Aotearoa New Zealand up to 2040 for the total population and Māori population based on Statistics New Zealand’s population projections for median population growth and immigration. If current trends continue, by 2030 there will be an additional 92 cases of EOCRC diagnosed per year, this number rising to an additional 255 EOCRC cases per year in 2040, resulting in a total number of predicted cases of EOCRC of 524. Due to the rapidly aging population, we are also likely to see large increases in total case numbers diagnosed over the age of 80 years, with an additional 465 cases diagnosed in 2030 compared to 2020, representing 34.5% of all CRC cases, and potentially a further 1029 cases per year by 2040, representing 43% of all CRC diagnosed. For Māori, by 2040, the total number of cases of EOCRC is predicted to more than double, and make up 20.55% of all CRC cases (Table 4).

**Table 4:** Projections to 2040

		2020 actual		2030 predicted			2040 predicted		
		*count*	*%*	*count*	*%*	*(CI)*	*count*	*%*	*(CI)*
**Total Ethnicity**									
	Population	5.1 M		5.4 M			5.8 M		
	Cases	3142		3665		(3581, 3749)	4255		(4099, 4411)
	Incidence	61.8		67.36		(65.81, 68.90)	73.07		(70.39, 75.75)
EOCRC	Population	3.4 M	65.90%	3.4 M	62.95%		3.5 M	60.31%	
	Cases	269	8.56%	361	9.85%	(331, 391)	524	12.31%	(458, 590)
	Incidence	8.00		10.50		(9.7, 11.4)	14.90		(13.0, 16.8)
Older 80+	Population	0.2 M	3.65%	0.3 M	5.29%		0.4 M	7.14%	
	Cases	801	25.49%	1266	34.54%	(1210, 1322)	1830	43.01%	(1711, 1949)
	Incidence	431.76		440.04		(420.7, 459.4)	439.96		(411.4, 468.5)
**Māori ethnicity**									
	Population	854.9 K		1,019.30 K			1,187.6 K		
	Cases	244		327		(308, 346)	438		(400, 476)
	Incidence	28.54		32.04		(30.22, 33.94)	36.88		(33.68, 40.08)
EOCRC	Population	678.78 K	77.34%	788.40 K	77.35%		897.10 K	75.5%	
	Cases	43	17.6%	52	15.90%	[44, 60]	90	20.55%	(70, 109)
	Incidence	6.33		6.64		(5.63, 7.64)	10		(7.84, 12.17)
Older 70+	Population	33.03 K	3.86%	60.30 K	5.92%		97.60 K	8.22%	
	Cases	69	28.3%	130	39.76%	(117, 142)	201	45.89%	(173, 229)
	Incidence	208.9		215.19		(194.45, 235.94)	205.61		(176.76, 234.45)

Predicted case numbers and incidence with 95% confidence intervals (in parentheses) based on Poisson regression on Median population growth projections for 2030 and 2040. Actual data shown for 2020

All model terms were tested for significance with likelihood ratio tests; no other interactions significantly improved fit using the likelihood ratio test (*P* > 0.05). The models showed no evidence of lack of fit, *P* > 0.408, (0.269 Māori), over dispersion or inferior fit to a Negative Binomial model, Log Likelihood test df = 29, *P* = 0.795.(df = 18 *P* > 0.99). Autocorrelation plots revealed no evidence of correlation with lagged variables (all *r* < 0.5.)

## Discussion

This study has shown that the age-standardized incidence of CRC is falling, while the incidence of EOCRC in Aotearoa New Zealand is continuing to rise. This increase in EOCRC incidence is predominantly associated with distal colonic and rectal cancers, with smaller increases seen in proximal colonic cancers. In patients aged 50–79, however, there are ongoing decreases in CRC incidence across all sites. Concerningly, in contrast to the total population, crude incidence of CRC in Māori is rising, driven by increases in incidence of EOCRC in Māori patients.

Comparing these results to data published by Gandhi et al [2] that described incidence rates in Aotearoa New Zealand from 1995 to 2012, rates of increase of EOCRC appear to be rising. The rates of increase of rectal cancer incidence in women is accelerating from 13% per decade to 25% per decade, while rates of increase in distal colonic cancer in men has risen from 14% per decade to 35% per decade, and likewise rates of increase of rectal cancer incidence in men has risen from 18% per decade to 32%. This study has also shown statistically significant increases in other sites that previously failed to reach significance, with rates of distal colonic cancers in women rising by 29% per decade (*p* = < 0.0005), and rates of proximal colonic cancers in women rising by 14% (*p* = < 0.05) and 20% in men (*p* = < 0.005). Our modelling predicts that if these trends continue to 2030, there will be 361 cases of EOCRC diagnosed in Aotearoa New Zealand, an increase of 92 cases per year when compared with numbers seen in 2020. These numbers increase to a potential 524 total cases per year by 2040, an additional 255 cases of EOCRC per year.

Our data shows that early onset colorectal cancers are predominately sigmoid and rectal cancers as has been described in other studies [2, 16]. This is, however, the first data to also show significant increases in proximal colonic cancers here in Aotearoa New Zealand. This is consistent with trends seen in Europe over the past 25 years [17]. The evolving pattern of disease will have significant implications with regards to potential screening methods, with proximal cancers being missed by flexible sigmoidoscopy.

While a recent metanalysis found that there was no material difference in cancer specific survival in EOCRC compared to older patients [7], strategies are needed to help improve outcomes in EOCRC. One issue affecting EOCRC internationally is delay to diagnosis [5, 18], with delays contributing to more advanced stage at diagnosis, and resulting in poorer outcomes [19]. These delays may be driven by patients not seeking medical advice for symptoms, or doctors not appreciating the epidemiological shift in the pattern of this disease and, therefore, not investigating young patients appropriately. One study found that the largest contributor to delays in diagnosis in rectal cancer in patients under 50 years was patients not seeking medical advice, with a median time from symptom onset to seeing a doctor of 121 days, compared to only 21 days in patients over 50 years [18]. Improving patient education about when to seek medical attention may help improve these delays. Bowel Cancer New Zealand’s recent campaign, ‘Never too young’, is an example of an organisation helping to improve patient education about what signs to look out for [20]. Likewise, improving physician education to ensure timely workup of symptomatic patients once they have presented is also crucial. There is no specific data on whether there is different survival in EOCRC in Aotearoa New Zealand or on delays to diagnosis in those aged under 50 years in Aotearoa New Zealand, and these are both areas where further research would be beneficial.

Lowering the age of screening will also help, through both prevention of some cancers and earlier diagnosis of others. Aotearoa New Zealand has only recently started screening patients over 60 years of age, and the exact impact of this in the screened population has yet to be established. In contrast to many other countries around the world that have been screening people over the age of 50 years, including Canada, UK, and Germany, with the USA and Australia recently screening from age 45 years [21–24]. Several American guidelines, including the American Cancer Society (ACS) [25], National Comprehensive Cancer Network (NCCN) [26], American College of Gastroenterology (ACG) [27] and the US preventative task force now recommend starting screening of average risk individuals at 45 years of age [28, 29]. While there is no direct evidence that screening below the age of 50 years will reduce EOCRC, countries such as Italy, Austria and Japan have been screening patients in their 40’s since the 1980s [24], and they are some of the few countries internationally where EOCRC rates have been declining [3, 17, 30, 31]. 

We acknowledge that there will be issues with resourcing, and health systems need to balance providing access to colonoscopy for screening programmes, without increasing wait times for at-risk or symptomatic populations. This, however, should not limit goals for standards of care from being set, and increasing endoscopy capacity should be a priority for health systems to reach these goals. In the long term, it will save the health system money due to reduced treatment costs as a result of earlier diagnosis of CRC. Modelling studies from both USA and Canada have shown screening from 45 years, or as low as 40 years to be cost effective [32, 33]. Because of this, we believe that screening for average-risk individuals in Aotearoa New Zealand should start at age 45. It is important to note that while this will only reduce the impact in those eligible for screening, this makes up a large proportion of all EOCRC, with 44% of all EOCRC diagnosed during our study period occurring in those aged 45–49 years (see Supplementary Table 4). Looking forward, as methods of diagnosis improve, such as through the development of biomarkers, screening could potentially selectively target younger higher-risk patients [34]. Timely diagnosis of younger symptomatic individuals is also critical. Given that the largest increases in EOCRC incidence were seen in distal colonic and rectal cancers, clinicians may consider flexible sigmoidoscopy as a tool to investigate per rectal bleeding in young patients.

This study has observed increases in the incidence of CRC in Māori, with this change being driven by increases in EOCRC. Additionally, Māori patients aged 50–69 years are not seeing the same decreases in incidence rates that are occurring in the total population. Ongoing sequelae of colonisation includes reduced life expectancy in Māori compared to non-Māori; [35] hence, to keep proportions comparative, the age threshold for older Māori was reduced to 70 years [36]. While incidence rates of CRC in Māori patients remain lower than in non-Māori, due to the different age distribution of the Māori population, with a far higher proportion of younger people, Māori patients are disproportionately affected by early-onset disease, with 30% of diagnoses in Māori women and 25% in Māori men occurring prior to the age of 50 years [37]. Also, Māori, once diagnosed with CRC are more likely to die from their disease than non-Māori [38]. Causes for this are likely multifactorial and there is evidence of inequitable access to early diagnosis, chemotherapy, and treatment in CRC [39]. Māori are also more likely to be diagnosed with stage 4 disease (stage 4 colon cancer 31.6% vs. 22.8%, stage 4 rectal cancer 29.4% vs. 18.1%) [40], which may be due to greater delays to diagnosis. These factors make the rate of increase in Māori patients under 50 extremely concerning and if these increases go unchecked, we will see rates in Māori overtake those of the general population. To help combat this inequity, lowering of the screening age in Aotearoa New Zealand should be extended further for Māori. The fact that CRC incidence in Māori aged 50–69 years is not falling, may also be a result of inequitable access to health interventions, such as bowel cancer screening, or increasing use of diagnostic colonoscopy for symptomatic patients, which has previously been credited with helping drive the decreases in incidence we are seeing in older adults [2]. 

The factors driving the increase in EOCRC incidence are unknown but are likely to be multifactorial. LOCRC appears to be driven by the interaction of an individual’s microbiome and diet, with conventional risk factors, such as obesity, alcohol, processed meat, sugary drinks, and a ‘Western diet’ (high fat, high meat, and low fibre), and such mechanisms are likely with EOCRC [41–47], However the mechanism must be different from LOCRC to be driving different changes in incidence. While several bacterial species have already been implicated in adenoma or LOCRC development [48–50], data specific to EOCRC suggest that the microbiome in patients with EOCRC is different compared that found in patients with LOCRC and healthy controls [51]. These differences may reflect early-life events and/or ongoing environmental factors, many of which emerged over the past several decades. These include caesarean delivery [52], formula feeding [53], antibiotic use [54], changing diet, synthetic food dyes, MSG high-fructose corn syrup, or microplastics [55]. 

Managing the impact that cancer diagnosis and treatment has on younger patients also needs to be considered. Recent research has shown that there are large deficits in cancer survivorship care in Aotearoa New Zealand, this having profound impacts on the wellbeing of cancer patients after treatment [56]. This is particularly important in EOCRC patients, with research showing psychosocial impacts being more pronounced in EOCRC compared with LOCRC [57]. This is another area where we believe more focus and funding is critical to improve the quality of life of the increasing numbers of EOCRC patients.

It is however worth emphasising that despite the rising incidence of EOCRC, CRC remains largely a disease of older adults. From 2015 to 2020, an average of 92.5% of CRC was diagnosed in those over the age of 50 years and the absolute incidence rates of EOCRC are a small fraction of those seen in older patients (Table 1). It is also worth considering that for patients over the age of 80 years, the increasing incidence rates may be a result of increased detection, rather than a true increasing prevalence of disease, e.g., increasing incidental findings of CRC picked up on cross sectional imaging for another indication [58]. Whatever the cause, due to the rapidly aging population the volume of CRC diagnoses in patients over 80 years is set to increase dramatically. Our modelling has shown that the number of CRC cases diagnosed over the age of 80 will increase from 801 in 2020 to 1266 as close as 2030, and to a potential 1830 cases per year in 2040. This will mean that CRCs diagnosed over the age of 80 years may account for 34.5% of all CRC cases in 2030, and 43% in 2040. How to manage this will be another huge challenge ahead for our already struggling health system. Discussions around whether this may necessitate rationing of health-care resources for elderly people are already underway, and is a contentious issue that is likely to become more urgent [59–61]. 

A notable strength of this study is that it is the most up-to-date, population-based data describing overall trends in CRC incidence in Aotearoa New Zealand by age, sex, and site of disease. From 1995 it has been compulsory to register all new cancer diagnoses in Aotearoa New Zealand with the New Zealand Cancer Registry, so we can be confident that this study captures the vast majority of new diagnoses of colorectal adenocarcinoma in Aotearoa New Zealand. There are, however, some limitations, namely that cancer registrations can be missed or miscategorised, either with an incorrect type or site of disease; these are problems inherent with using any registry data. The impact of bowel cancer screening has also not been specifically addressed in this paper. It was trialled in one area of Aotearoa New Zealand starting from 2011, and then rolled out nationally from 2019 to 2021. Therefore, it will currently be having an impact on CRC incidence rates in the middle age bracket. There are well-documented problems with undercounting of Māori ethnicity [62] and there was also a change to Māori ethnicity classification in 2006, which may influence calculated incidence in this analysis, and result in underestimation of incidence rates. This research has been approved and guided by input from Māori researchers, and following the steps outlined in the CONSIDER statement [14], however none of the researchers are themselves Māori and we acknowledge this as a limitation. While every care has been taken with the methodology, we urge caution when interpreting these projections as they are sensitive to changes in population structure, due to extrinsic factors, such as, immigration and economic circumstances. For our predictions, we have assumed an ongoing linear trend of all incidence rates, but for EOCRC in recent years, the rate of increase has been increasing, so our predictions in this group may be an underestimation. This also does not take into account any impact of screening, which is likely to reduce the incidence in CRC in older populations. There is currently no data on EOCRC survival in Aotearoa New Zealand and we acknowledge that this as an area where further research is required.

## Conclusion

The incidence of EOCRC in Aotearoa New Zealand continues to increase, and incidence rates are rising faster in Māori compared to the total population. More research is required to establish the causes of this trend and develop strategies to reduce incidence and determine the best management and support for these patients. Additionally with an aging population, CRC diagnosis in the oldest age groups will present a significant and increasing burden to health systems.

### Electronic supplementary material

Below is the link to the electronic supplementary material.

Supplementary Material 1

## Data Availability

The datasets used and/or analysed during the current study are available from the corresponding author on reasonable request, or by request from the New Zealand Cancer registry.
