# Supplementary material for: The incidence of early onset colorectal cancer in Aotearoa New Zealand: 2000–2020
Source: BMC Cancer. 2024 Apr 12;24:456. doi: 10.1186/s12885-024-12122-y (PMC11010297; doi:10.1186/s12885-024-12122-y)
Supplement: Supplementary file 1 — Supplementary Material 1 [file 12885_2024_12122_MOESM1_ESM.docx]

**Supplementary table 1:** Consolidated Criteria for Strengthening Reporting of Health Research Involving Indigenous Peoples (CONSIDER) checklist**.**

| **Governance** | |
| --- | --- |
| 1. | *Describe partnership agreements between the research institution and Indigenous-governing organization for the research, (e.g., Informal agreements through to MOU (Memorandum of Understanding) or MOA (Memorandum of Agreement)).*   - MOU between Te Rūnanga o Ngāi Tahu and the University of Otago |
| 2. | *Describe accountability and review mechanisms within the partnership agreement that addresses harm minimization.*   - All requirements of Human Ethics (Health) review met. - Rangahau Māori Plan and Māori health advancement reviewed and endorsed by UOC Māori Research Expertise Rōpū |
| 3. | *Specify how the research partnership agreement includes protection of Indigenous intellectual property and knowledge arising from the research, including financial and intellectual benefits generated (e.g., development of traditional medicines for commercial purposes or supporting the Indigenous community to develop commercialization proposals generated from the research).*   - The results of this research are descriptive in nature, indigenous governance rights are protected by the University of Otago’s commitment to Te Tiriti o Waitangi affirmed in the MOU. |
| **Prioritization** | |
| 4. | *Explain how the research aims emerged from priorities identified by either Indigenous stakeholders, governing bodies, funders, non-government organization(s), stakeholders, consumers, and empirical evidence.*   - There is considerable evidence regarding Māori health inequities that with regards to colorectal cancer, including barriers to access to treatment and poorer outcomes. - In particular early onset bowel cancer affects a greater proportion of Māori. - This research provides fundamental information to allow equity in policy decisions, for example evidence that age of bowel screening should be lowered for Māori. |
|  | **Relationships** (Indigenous stakeholders/participants/research team) |
| 5. | *Specify measures that adhere and honor Indigenous ethical guidelines, processes, and approvals for all relevant Indigenous stakeholders, recognizing that multiple Indigenous partners may be involved, e.g., Indigenous ethics committee approval, regional/national ethics approval processes*.   - All requirements of Human Ethics (Health) review met. - Rangahau Māori Plan and Māori health advancement reviewed and endorsed by UOC Māori Research Expertise Rōpū (UOC MRER) |
| 6. | *Report how Indigenous stakeholders were involved in the research processes (i.e., research design, funding, implementation, analysis, dissemination/recruitment).*   - Ongoing relationship between Ngāi Tahu and UOC managed by UOC MRER - Research design and implementation informed by University of Otago Maori Health Advancement courses 100/200/300 run by indigenous academics. |
| 7. | *Describe the expertise of the research team in Indigenous health and research*   - All guidance from UOC MRER has been acted on and all researchers are actively engaged in Māori Health Advancement CPE. - A/Prof John Pearson has published on Multiple Sclerosis epidemiology in Māori with guidance from UOC Māori Research Advisor, is currently working on cardiac risk led by a Pasifika researcher, and has participated in a introduction to history and tikanga at Tūāhuriri Marae. - Prof Frizelle has been involved several Hauroa Ora and Marae-based community education huis about colorectal cancer. - Dr Purcell and Dr Glyn have participated in a Tane Ora day at Hakatere Marae, organized through Nikki Ross, a Māori Clinical Research Nurse working on CRC projects in the Department of Surgery and Clinical Care. This is part of the foundation for establishing patient-led research within the Department. |
|  | **Methodology** |
| 8. | *Describe the methodological approach of the research including a rationale of methods used and implication for Indigenous stakeholders, e.g., privacy and confidentiality (individual and collective)*   - All modelling and results on Māori performed separately with models appropriate to their data. - All results presented at summary level only to ensure individual and collective privacy - Prioritized ethnicity used to include all those with Māori ethnicity. - Age groups adjusted to give equitable comparison for older Māori. - Undercount of Māori acknowledged as a limitation. |
| 9. | *Describe how the research methodology incorporated consideration of the physical, social, economic and cultural environment of the participants and prospective participants. (e.g., impacts of colonization, racism, and social justice). As well as Indigenous worldviews.*   - The Māori population in Aotearoa has a very different age distribution when compared to the total population, so we have accounted for this in our analysis. We have adjusted the cut off of our ‘older’ group in Māori to be above 70 years in comparison to above 80 years in the total population, in both groups this cut off results in 3% of the respective population. - Impacts of colonization, racism and social justice referenced in discussion. |
|  | **Participation** |
| 10. | *Specify how individual and collective consent was sought to conduct future analysis on collected samples and data (e.g., additional secondary analyses; third-parties accessing samples (genetic, tissue, blood) for further analyses).*   - No tissues required, all data collected by national agencies under their own ethics and Acts. - All data is consider Tāonga and treated accordingly. |
| 11. | *Described how the resource demands (current and future) placed on Indigenous participants and communities involved in the research were identified and agreed upon including any resourcing for participation, knowledge, and expertise.*   - No resource demands were placed on any Māori participants in order to complete this research |
| 12 | *Specify how biological tissue and other samples including data were stored, explaining the processes of removal from traditional lands, if done, and of disposal.*   - Data accessed was summary data only and was not identifiable. It was stored on a secure system with all access logged and requiring password authentication limited to administrative and research staff only. |
|  | **Capacity** |
| 13. | *Explain how the research supported the development and maintenance of Indigenous research capacity (e.g., specific funding of Indigenous researchers).*   - Research was undertaken by the Department of Surgery and Critical Care, UOC. While no Māori researchers were directly involved with the undertaking of this research, this department frequently supports Māori students through summer studentships, and supports research undertaken by Māori registrars throughout the department. The Department has recently employed a Māori Clinical Research Nurse to specifically work on CRC projects involving Māori patients and whānau. |
| 14. | *Discuss how the research team undertook professional development opportunities to develop the capacity to partner with Indigenous stakeholders?*   - Research design and implementation informed by University of Otago Maori Health Advancement courses 100/200/300 run by indigenous academics. Dr Oliver Waddell has complete courses at 100 level, Dr Rachel Purcell and A/Prof John Pearson have completed courses at 200 level and currently engaged in 300 level training. - All researchers actively engages in Te Reo Māori mahi (Māori language training), Dr Waddell has passed Te Kaupae level 2 and is currently studying Te Reo Te Kaupae level 3 Ara institute of technology |
|  | **Analysis** **and** **interpretation** |
| 15. | *Specify how the research analysis and reporting supported critical inquiry and a strength-based approach that was inclusive of Indigenous values.*   - Māori data analysed separately, adjusted to the Māori population distribution and with models and with appropriate models and age groups. Interpretation informed by Te Ao Māori perspectives imparted from indigenous instructors. |
|  | **Dissemination** |
| 16. | *Describe the dissemination of the research findings to relevant Indigenous governing bodies and peoples.*   - Production of Māori specific epidemiology in relevant media is critical to Māori self-determination and health advancement. Results will be published in academic journals, media releases and public hui. |
| 17. | *Discuss the process for knowledge translation and implementation to support Indigenous advancement (e.g., research capacity, policy, investment).*   - We believe our findings can help guide recommendations for policy changes which could benefit Māori communities and help address the inequities present. For example, we aim to continue to fight for lowering of the bowel cancer screening age in the general population and argue that any lowering should be extended for Māori to help address the inequities present. Citing this research will help guide those recommendations. |

**Supplementary table 2:** Total population incidence rates by year, age, sex and site of disease.

| **Incidence per 100,000** | | | | | |
| --- | --- | --- | --- | --- | --- |
|  | 2000 | 2005 | 2010 | 2015 | 2020 |
| **Age <50 years** |  |  |  |  |  |
| Women |  |  |  |  |  |
| Proximal | 1.64 | 1.29 | 1.58 | 1.56 | 2.24 |
| Distal | 1.92 | 1.5 | 1.78 | 1.5 | 2.84 |
| Rectal | 1.78 | 2.18 | 2.89 | 2.54 | 2.9 |
| Men |  |  |  |  |  |
| Proximal | 0.71 | 1.02 | 1.33 | 1.44 | 2.42 |
| Distal | 0.92 | 1.16 | 1.66 | 1.5 | 2 |
| Rectal | 1.85 | 2.39 | 2.59 | 3.2 | 3.71 |
| **Age 50**–**79 years** |  |  |  |  |  |
| Women |  |  |  |  |  |
| Proximal | 76.81 | 71.16 | 66.47 | 55.21 | 44.23 |
| Distal | 42.55 | 42.17 | 35.11 | 25.2 | 28.19 |
| Rectal | 55.11 | 44.43 | 37.23 | 37.51 | 33.71 |
| Men |  |  |  |  |  |
| Proximal | 61.78 | 60.44 | 57.45 | 44.02 | 45.82 |
| Distal | 60.2 | 52.14 | 52.45 | 50.83 | 52.22 |
| Rectal | 90.3 | 75.64 | 68.14 | 66.57 | 65.54 |
| **Age 80+ years** |  |  |  |  |  |
| Women |  |  |  |  |  |
| Proximal | 209.25 | 227.03 | 275.09 | 252.26 | 249.77 |
| Distal | 96.14 | 79.7 | 66.33 | 79.93 | 68.71 |
| Rectal | 80.59 | 112.31 | 100.03 | 88.24 | 85.42 |
| Men |  |  |  |  |  |
| Proximal | 177.98 | 202.15 | 241.74 | 233.67 | 205.5 |
| Distal | 134.17 | 103.23 | 98.74 | 133.31 | 122.01 |
| Rectal | 158.82 | 154.84 | 160.03 | 161.77 | 147.7 |

**Supplementary table 3:** Māori population incidence rates by year age, sex and site of disease.

| **Incidence per 100,000 in Māori** | | | | | |
| --- | --- | --- | --- | --- | --- |
|  | 2000 | 2005 | 2010 | 2015 | 2020 |
| **Age <50 years** | | | | | |
| Women | | | | | |
| Proximal | 0.38 | 0.72 | 1.06 | 0.34 | 1.49 |
| Distal | 1.13 | 1.08 | 1.06 | 1.02 | 1.49 |
| Rectal | 0.75 | 0.72 | 2.84 | 1.02 | 2.08 |
| Men | | | | | |
| Proximal | 0 | 0.73 | 0.36 | 1.05 | 2.63 |
| Distal | 0.76 | 0 | 1.08 | 0.35 | 2.63 |
| Rectal | 0.38 | 1.46 | 2.53 | 3.86 | 2.34 |
| **Age 50–70 years** | | | | | |
| Women | | | | | |
| Proximal | 26.83 | 35.79 | 19.79 | 25.51 | 22.76 |
| Distal | 13.41 | 16.52 | 24.19 | 22.11 | 18.74 |
| Rectal | 33.53 | 24.78 | 32.99 | 20.41 | 26.78 |
| Men | | | | | |
| Proximal | 17.76 | 20.72 | 36.49 | 23.18 | 35.08 |
| Distal | 21.31 | 23.68 | 38.92 | 28.98 | 32.16 |
| Rectal | 46.18 | 62.15 | 36.49 | 50.23 | 51.16 |
| **Age 70+ years** | | | | | |
| Women | | | | | |
| Proximal | 46.22 | 101.65 | 125.85 | 90.91 | 60.57 |
| Distal | 46.22 | 50.83 | 58.08 | 37.88 | 38.55 |
| Rectal | 92.45 | 50.83 | 58.08 | 60.61 | 55.07 |
| Men | | | | | |
| Proximal | 82.14 | 147.54 | 48.13 | 47.66 | 80.7 |
| Distal | 164.27 | 81.97 | 72.2 | 47.66 | 107.6 |
| Rectal | 102.67 | 147.54 | 60.17 | 85.8 | 87.42 |

**Supplementary table 4.** Proportions of cases diagnosed under the age of 50 by different age brackets.

|  | 2000 | | | | 2010 | | | | 2020 | | | | Total study period (2000-2020) | | | |
| --- | --- | --- | --- | --- | --- | --- | --- | --- | --- | --- | --- | --- | --- | --- | --- | --- |
|  | Total population | | Māori population | | Total population | | Māori population | | Total population | | Māori population | | Total population | | Māori population | |
| Age group | n | %* | n | %* | n | %* | n | %* | n | %* | n | %* | n | %* | n | %* |
| <30 years | 4 | 3.2% | 0 | 0.0% | 9 | 5.7% | 0 | 0.0% | 12 | 4.4% | 2 | 4.7% | 199 | 5% | 28 | 6% |
| 30-39 years | 26 | 21.0% | 3 | 27.3% | 26 | 16.4% | 10 | 37.0% | 78 | 28.9% | 9 | 20.9% | 924 | 24% | 125 | 28% |
| 40-44 years | 30 | 24.2% | 3 | 27.3% | 42 | 26.4% | 6 | 22.2% | 85 | 31.5% | 15 | 34.9% | 1005 | 27% | 123 | 28% |
| 45-49 years | 64 | 51.6% | 5 | 45.5% | 82 | 51.6% | 11 | 40.7% | 95 | 35.2% | 17 | 39.5% | 1645 | 44% | 167 | 38% |
| Total | 124 |  | 11 |  | 159 |  | 27 |  | 270 |  | 43 |  | 3773 |  | 443 |  |

*percentages are as a proportion of all cases under 50 years in that population
